# Modulation of sperm function by estrogen and progesterone using in vitro experiments: A systematic review

**DOI:** 10.1371/journal.pone.0330009

**Published:** 2025-08-11

**Authors:** Muloongo C. Sitali, Madalitso Chelenga

**Affiliations:** 1 Department of Biomedical Sciences, School of Veterinary Medicine, The University of Zambia, Lusaka, Zambia; 2 Department of Veterinary Clinical Studies, Faculty of Veterinary Medicine, The Lilongwe University of Agriculture and Natural Resources, Lilongwe, Malawi; King Saud University / Zagazig University, EGYPT

## Abstract

**Background:**

The steroid hormones progesterone (P4) and Estrogen (E2) are essential for reproduction in both male and female mammals. Steroid hormones can alter sperm function *in vitro* via several mechanisms, thereby facilitating fertilization of female germ cells. The purpose of this systematic review is to investigate the *in vitro* experimental models that are used to understand the functionality of sperm after receiving supplementation with E2 and P4 in samples obtained from animal species using sperm oviductal epithelial cells (OECs) co-cultures and cultures of sperm in culture media only without OECs.

**Methodology:**

A systematic literature search was conducted using Web of Science, Google Scholar, and PubMed to examine *in vitro* experimental models involving E2 and P4, aiming to understand sperm functionality in animals with or without OECs in culture media. A total of 32 out of 660 retrieved articles met the inclusion criteria, from which data were extracted, synthesized, and presented in a descriptive format.

**Results:**

Sperm and oviducts from hamsters, mice, rats, monkeys, porcine, ovine, and bovine were used. Steroids E2 and P4 were administered at varying doses, either individually or in combination, with several studies observing dose-dependent effects on sperm function. In sperm-OECs co-cultures, P4 consistently promoted sperm hyperactivation, capacitation, and acrosome reaction (AR) via CatSper channel mechanisms, facilitating sperm release from OECs. Contrastingly, E2 delayed capacitation while enhancing sperm binding to the OECs. Similarly, supplementation of P4 in culture media alone without OECs induced sperm AR and capacitation, altered sperm motility, and improved *in vitro* fertilization (IVF) rates. Variations in motility and the binding ability of sperm to the isthmus and ampulla were reported in some studies.

**Conclusion:**

The *in vitro* studies revealed that E2 and P4 alter sperm behavior dependent on dose, and improve sperm fertilizing ability in IVF programs across species. Future studies should focus on investigating and clarifying the species-specific sperm mechanisms following steroid hormone supplementation to enhance our understanding and improve the assisted reproductive technology outcomes.

## 1. Introduction

The mammalian spermatozoa serves a single purpose for their existence, *i.e*., to deliver half of the required number of chromosomes for oocyte activation necessary to initiate embryonic development. Many sperm are released during ejaculation. However, only a minuscule number can reach the site of fertilization located in the ampulla region of the oviduct [1]. Successful fertilization requires that the spermatozoa and oocyte meet at the right place and time in the oviduct, within 12–24 h after ovulation [2,3], where only the best spermatozoa can successfully fertilize the oocyte [2,4] with minimal chances of polyspermy [5,6]. However, the sperm released at the time of ejaculation are incapable of fertilizing the oocyte and must reside in the female reproductive tract before they acquire the capacity to complete the fertilization process [6]. Moreover, sperm in the female reproductive tract are considered foreign cells, which might affect their longevity due to immunological surveillance [7]. Therefore, the female reproductive tract, particularly the oviduct, must be well adapted to achieve sperm transport, storage, survival, and sperm acquisition of fertilization competence (which includes, but is not limited to, capacitation and hypermotility) in the female reproductive tract.

The oviduct is a tube-like structure that connects the ovary to the uterus [8,9]. This structure is composed of the infundibulum, which captures the oocyte after ovulation; the ampulla, which contains large numbers of ciliated epithelial cells and is the site of fertilization; and the isthmus, which also contains a large number of secretory epithelial cells [9], and is a site where sperm are stored waiting for the ovulation signal. The oviductal function is mediated by oviductal fluids that provide nutrients and enzymes with anti-oxidant effects [10–12], as well as the function of the oviductal epithelial cells (OECs) that mediate the sperm-oviductal interactions [13,14]. The OECs in the isthmus region contribute to sperm storage function in the female reproductive tract by adhering to sperm, thereby forming the functional sperm reservoir where sperm are stored before ovulation [6].

The formation of the sperm reservoir is further essential for sperm selection, maintaining the viability of the sperm while inhibiting premature capacitation [3,4,6,14–16]. When the time is right, the sperm stored in the reservoir is released and transported to the site of fertilization [17]. The oviductal environment ensures that the sperm reaches the fertilization site through mechanisms such as chemotaxis and/or thermotaxis [18] to direct them to the ampulla. During movement or storage, the sperm are modified by the binding of different oviductal proteins (such as osteopontin) to increase sperm viability, motility, and capacitation [19]. Furthermore, it has been reported that the oviduct can also sense the presence of sperm and adjust the protein content and antioxidants in the oviductal fluid to protect sperm from oxidative stress and other functionalities [20]. Given these oviductal functions, it is apparent that dysregulation or disruption of the oviductal environment and function can result in failed fertilization, embryonic loss, and other possible life-threatening conditions such as ectopic pregnancy [9].

Understanding how the oviductal environment is regulated to successfully achieve the aforementioned functionalities is very crucial to the treatment of some forms of infertility and applications in assisted reproductive technologies (ARTs). It has been reported that the changes in progesterone (P4) and estrogen (E2) levels have a significant contribution to the mechanisms that govern the release of sperm from the functional sperm reservoir, sperm kinematics, and capacitation [21–24]. Furthermore, it has been demonstrated that smooth muscle contraction in the oviduct is regulated by prostaglandins through prostanoid receptors, which are modulated by E2 [25]. Besides, the mechanisms of action of E2 and P4 on sperm function primarily involve modulating the expression of specific receptors on sperm cells [26], which in turn affects sperm physiology, including motility, capacitation, and interaction with the female reproductive tract [27,28]. Even though ARTs, particularly *in vitro* fertilization (IVF), and sex sorting have enjoyed considerable success, the quality of embryos produced *in vitro* is lower than *in vivo*-derived counterparts. Furthermore, IVF has been associated with certain abnormalities. Notably, the large offspring syndrome has been observed in bovine and ovine offspring following the transfer of *in vitro*-produced embryos [29–31]. Other ARTs, such as semen sorting still succumb to poor outcomes despite recent intensive research aiming at improving the outcome of associated protocols. For instance, previous studies demonstrated that adding oviductal proteins to sperm diluents improves the fertilization ability and survival of sex-sorted sperm [32,33]. This suggests that a deeper understanding of the detailed molecular mechanism involved in sperm functionalities including the changes in the oviductal fluid composition and the transcriptome mediated by fluctuations in E2 and P4 concentrations during the estrous cycle or due to the presence of gametes [34] is essential for the improvement of ARTs protocols and solving certain fertility issues [6].

Several studies investigating the behavior and interaction of sperm with the oviduct have been conducted using *in vitro* models, essentially because the oviduct is a difficult-to-reach organ to substantially conduct experiments *in vivo* [35]*.* These *in vitro* models include co-culturing the sperm with or without OECs [1,17] or using the whole oviduct *ex vivo* [36]. *In vitro,* P4 facilitates sperm hyperactivation and capacitation, processes that are necessary for successful fertilization [27]. Besides, pre-treating OECs with P4 optimizes the environment for sperm selection, mimicking *in vivo* conditions [37]. Accordingly, nanomolar concentrations of P4 were detected in the oviductal fluid ipsilateral to ovulation in cows and in both oviducts of sows around the time of ovulation [38,39]. Despite P4’s low levels at fertilization in many species [40–42], the hormone P4 remains critical for optimizing sperm function and fertilization competence *in vitro* [41,43,44]. In addition, to enhance the understanding of what happens *in vivo*, researchers can replicate the *in vivo* dynamic hormonal milieu that affects sperm function and fertilization by selecting oviducts at various stages of the estrus cycle to assess any potential oviduct stage influence on sperm binding to the OECs because the oviduct undergoes significant changes across the estrus cycle [15]. Therefore, this systematic review aims to, 1) consolidate the findings related to the outcomes of the *in vitro* studies that have been conducted to understand the effects of E2 and P4 on sperm behavior and interaction with or without the oviductal microenvironment, and 2) provide insight for future research necessary to further elucidate the oviductal function that could be used to treat certain forms of infertility and improve the outcomes of ARTs such as IVF and semen sorting in animal species.

## 2. Methods

For this systematic review, the Preferred Reporting Items for Systematic Reviews and Meta-Analyses (PRISMA) guidelines were followed [45]. The species selected, dose of E2 and P4, sample size, and mechanisms of action of the steroid hormones were systematically gathered, along with relevant study findings. According to PROSPERO’s rules, the *in vitro* study’s systematic review protocol could not be registered.

### 2.1 Eligibility criteria

#### 2.1.1 Inclusion criteria.

*In vitro* studies conducted between 1^st^ January 1980 and 30^th^ November 2024 were included following the PICOS (Population, Intervention, Comparison, Outcome, and Study design) framework as follows; (1) Population: different animal species from which semen, OECs or oviductal explants were obtained; (2) Intervention: E2 and P4 supplementation; (3) Comparison: sperm cultured in absence of E2 or P4 or other control set-ups; (4) Outcome: effect on acrosome reaction (AR), sperm kinematics, capacitation, hyperactivation, effect on sperm interaction with the OECs; and (5) Study design: all controlled *in vitro* experimental studies involving animal sperm co-cultures with or without OECs. The summary of the eligibility criteria is shown in Table 1.

**Table 1:** Inclusion and exclusion criteria summary.

Parameter	Inclusion criteria	Exclusion criteria
Article or study type	• *In vitro-*based original research studies	• Letters to the editor • Conference abstracts • Short communications or comments • Reviews and book chapters • Protocols • Case reports and case series • Encyclopedias on the subject
Publication period	• Studies conducted between 1^st^ January 1980–30^th^ November 2024	• Studies conducted before 1^st^ January 1980 of after 30^th^ November 2024
Study content	• Studies evaluating E2 and P4 supplementation *in vitro* experiments • *In vitro* studies using semen samples and OECs, explants, glycans or *ex vivo* oviducts from animal species • *In vitro* studies using semen samples from animal species and culture media only without OECs	• *In vitro* studies not using E2 and P4 supplementation • Studies are not *in vitro* experiments • *In vitro* experiments using semen and oviducts from humans, equines, and birds

### 2.2 Information sources

The information was obtained from the online databases namely; Web of Science, PubMed, and Google Scholar.

### 2.3 Search

To identify relevant studies in the electronic databases, a search strategy comprising appropriate keywords was constructed and applied to retrieve the articles. Search terms were used in the three databases between 4^th^ and 8^th^ December 2024. During the final stages of writing this article, a second search was performed on 20^th^ March 2025. The search phrase used read as follows; (17β estradiol supplementation OR estrogen* OR estradiol 17β supplementation OR estradiol 17β treatment OR progesterone* AND sperm survival* OR sperm viability OR sperm function*) AND (oviduct OR oviductal epithelial cell OR oviduct glycans) AND (acrosome reaction OR sperm capacitation OR sperm hyperactivation). A manual search of the reference section (back referencing) was done to locate possible articles that the automated search was unable to locate.

### 2.4 Study selection

**Fig 1:**
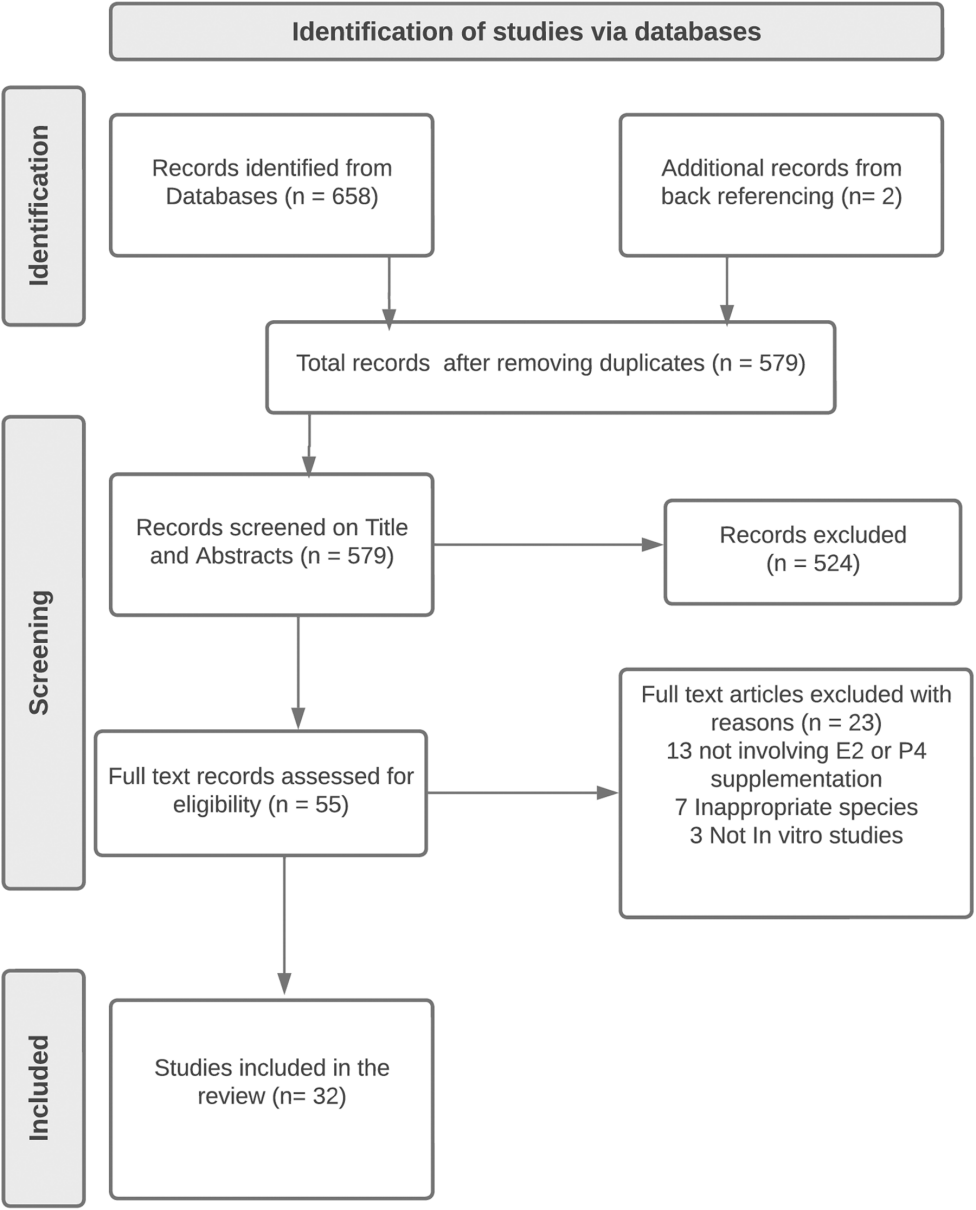
PRISMA flow diagram.

The screening process was conducted independently by the two reviewers to select relevant articles for the systematic review. First, we gathered 658 articles from the database search and two (2) articles from back referencing to give a total of 660 articles (Fig 1). Next, 81 duplicate articles were identified and removed. In the third step, 579 articles were screened on titles and abstracts to focus on studies assessing E2 and P4 supplementation to assess sperm function in sperm-OECs co-cultures and culture media without OECs and their impacts on IVF. Finally, a full-text assessment was performed to determine inclusion based on relevance to the study. 32 articles were selected that serve as the foundation for data extraction and narrative synthesis.

### 2.5 Data collection process

We used the Excel spreadsheet for the data extraction process encompassing key study characteristics explained in the data items subsection. The data extraction and verification were carried out by the two reviewers. In cases of disagreement, a consensus was reached through discussion.

### 2.6 Data items

The following data was extracted: Title, objectives of the study, the primary outcome of the *in vitro* experiments, the author and year of publication, mechanisms of action of the steroid hormones, the species employed, the E2 and P4 dosages, semen type, the sample size, and the estrus cycle stage of the oviduct section used.

### 2.7 Risk of bias assessment

**Fig 2:**
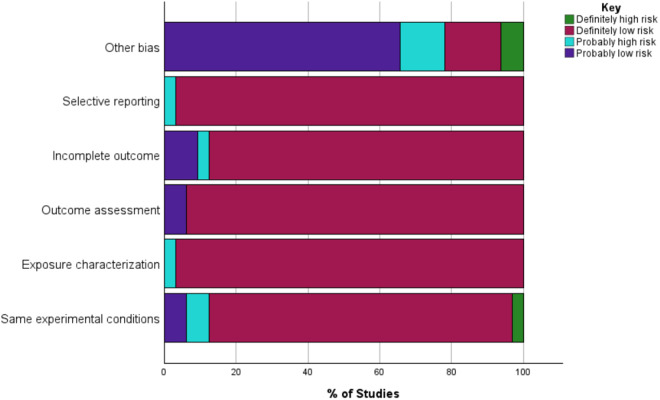
Risk of bias in included studies using the NTP-OHAT risk of bias tool.

The National Toxicology Program-Office of Health Assessment and Translation (NTP-OHAT) recommendations were followed in performing the risk of bias assessment [46]. The likelihood of bias in the included studies was evaluated by two independent reviewers. Risk of bias assessment categories for each included study were entered in an Excel spreadsheet as definitely high risk, definitely low risk, probably high risk, and probably low risk (Fig 2) and later exported to the Statistical Package for Social Sciences (SPSS) version 29 for analysis.

### 2.8 Quality assessment of the studies included

**Fig 3:**
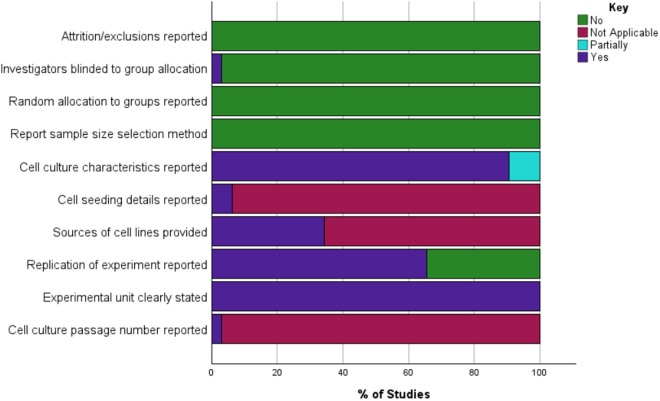
Quality assessment of the included studies.

Two reviewers independently evaluated the quality of the included studies using the checklist developed by [47] (Fig 3).

### 2.9 Proportions of species, semen type, oviductal epithelial cells, and steroid hormones used in the included studies

**Fig 4:**
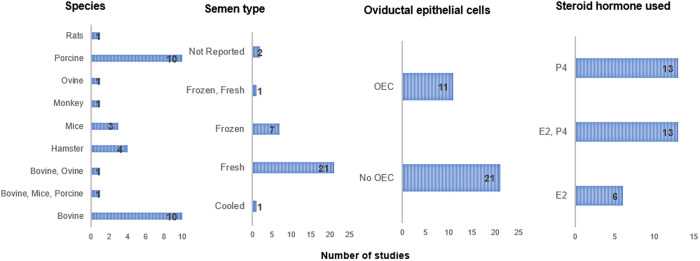
Charts showing the number of species, semen type, oviductal epithelial cells, and hormones used in the *in vitro* studies.

Estrogen and P4 have been used in several *in vitro* experiments to understand their effects on sperm functionality, in which the majority of the studies used fresh semen samples. 40% of the studies (i.e., thirteen studies) used a combination of E2 and P4, while 19% of the studies used E2, and thirteen studies used P4 treatment alone. Eleven studies used OECs co-cultures with spermatozoa from different animal species (i.e., porcine, bovine, and ovine) to understand the effects of E2 and P4 supplementation on sperm functionality *in vitro*, while twenty-one of the *in vitro* animal experiments used sperm samples from ovine, porcine, hamsters, monkey, bovine, mice, and rats and investigated the effects of E2 and P4 supplementation in culture media without OECs representing 66% of the included studies (Fig 4).

## 3. Oviduct region and estrous cycle stage of the oviducts used in the studies

**Fig 5:**
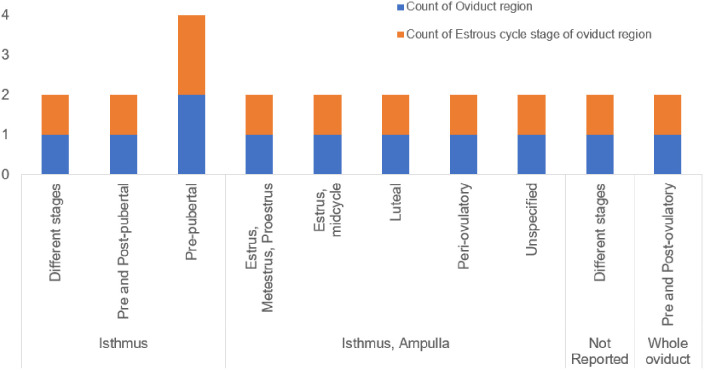
Oviduct regions and estrous cycle stage of the oviducts.

To assess sperm-OECs interaction, 46% of the included studies employed models incorporating both the isthmus and ampulla regions of the oviduct, obtained from animals at varying stages of the estrus cycle (Fig 5). In one study, it was reported that the binding ability of boar sperm is not influenced by the oviduct region used or by the estrus cycle stage [48]. Contrasting results were reported where a greater number of boar sperm bound to the isthmic than ampulla explants in the presence of estrus levels of steroids [49]. These differences could be attributed to the varying experimental conditions employed across the studies. Besides, there was a variation in the estrous cycle stage of the oviduct regions used in these studies.

## 4. Main study outcomes and synthesis of results

The effects of E2 and P4 supplementation on sperm behavior in the *in vitro* experimental models and the reported mechanisms of action in the studies are shown in Tables 2 and 3.

**Table 2:** Effects of E2 and P4 supplementation on sperm functionality using oviductal cells *in vitro.*

Species	Semen type	E2 and P4 effect on sperm	Mechanism of action	First Author
Porcine	Not Reported	P4 **⇈** capacitation	Does not interfere with the cytoskeleton dynamics and the cells doubling time	Cimini [37]
Porcine	Fresh	P4 **↝** sperm from OEC.	Active CatSper channels and proteasomal breakdown	Sharif [21]
Porcine	Fresh	P4 **↝** sperm from OEC	dependent on CatSper channels	Machado [22]
Bovine	Frozen	P4-induced sperm hyperactivation **↝** of sperm from OEC	Via non-genomic pathways by stimulating CatSper	Romero-Aguirregomezcorta [50]
Bovine/Ovine	Cooled	E2 and P4 **⇊** sperm kinematics and delays sperm capacitation	Not assessed	Alfradique [51]
Bovine	Frozen	P4 **↝** sperm from OEC⊗ by E2	Not assessed	Lamy [23]
Porcine	Fresh	P4 **↝** sperm from OEC than E2 treatment	Calcium mediated	Bureau [52]
Bovine	Frozen	E2 **⇈** ability of OEC to prolong motility	Not assessed	Boquest [53]
Bovine	Frozen	OECs **⇈** the survival and motility of sperm reliant on steroids	Not assessed	Lapointe [54]
Porcine	Fresh	E2 **⇈** binding of sperm to OEC	Not assessed	Suarez [48]
Porcine	Fresh	High E2 and low P4 **⇈** binding of sperm to OEC	Not assessed	Raychoudhury [49]

**Key:** **⇈** **Increases;** **⊗** **Inhibit;** **⇊** **Decreases;** **↝** **Cause release**

**Table 3:** Effects of E2 and P4 supplementation on sperm functionality without oviductal cells *in vitro.*

Species	Semen type	E2 and P4 effect on sperm	Mechanism of action	First Author
ICR Mice	Fresh	P4 ⇈ hyperactivation ⊗ by E2.	Via the membrane E2 receptor	Fujikura [55]
ICR Mice	Fresh	P4 ⇈ hyperactivation	Via the membrane P4 receptor of the sperm head	Suzuki [27]
Rats	Fresh	P4 ⇈ hyperactivation	Via the membrane P4 receptor regulated by phospholipase C, transmembrane adenylate cyclase and protein kinase A and non-genomic pathways	Miyazawa [56]
Ovine	Fresh	P4 and E2 ↟ ram sperm AR	P4 receptor and E2 (ERα and ERβ) receptors	Gimeno-Martos [28]
Bovine	Fresh	P4 ↟ AR in capacitated sperm	Via intact microtubules	Denisenko [57]
Porcine	Fresh	Steroids ↟ distribution of motility measurements in sperm subpopulations in a subtle manner	Not assessed	Ayala [58]
Porcine	Fresh	E2 ⇞⇟ overall motility, progressive motility, or the percentage of rapid motility.	E2 (ERα and ERβ) receptors	Gray [59]
Rhesus Monkey	Fresh	P4 ⇈ completion of capacitation.	CatSper Channel	Sumigama [44]
Bovine, ICR Mice, Porcine	Frozen, Fresh	E2, P4 ⇈ capacitation and AR.	Not assessed	Ryu [60]
Golden Hamster	Fresh	P4 ⇈ hyperactivation ⊗ E2. P4 and E2 ⇞⇟ on the percentage of motile spermatozoa.	Not assessed	Fujinoki [61]
Golden Hamster	Fresh	P4 ⇈ hyperactivation	Inositol 1,4,5-trisphosphate, Protein kinase C, and Protein kinase A signals	Fujinoki [62]
BALB/c Mice	Fresh	E2 ⇊ capacitation	Membrane receptors and tyrosine phosphorylation	Sebkova [63]
Golden Hamster	Fresh	P4 ⇈ hyperactivation but E2 ⇞⇟	Inhibition of tyrosine phosphorylation via non-genomic pathways	Fujinoki [64]
Bovine	Fresh	Lower dose E2 ⇈ motility and acrosome integrity. Higher dose ⇊ viability (sperm survival)	Not assessed	Ciftci [65]
Porcine	Not Reported	E2 ↟ capacitation of boar spermatozoa	Not assessed	Ded [66]
Golden Hamster	Fresh	P4 ⇈ hyperactivation	Non-genomic signal in relation to tyrosine phosphorylation and Phospholipase	Noguchi [67]
Bovine	Frozen	E2 stabilises the sperm plasma membrane. P4 ↟ capacitation and AR that are ⇊ by E2.	Not assessed	Lukoseviciute [68]
Bovine	Frozen	P4 ⇈ capacitation but ⇞⇟ on plasma membrane stability and AR in non-capacitated sperm	Not assessed	Lukoseviciute [69]
Bovine	Fresh	P4 has a concentration-dependent influence on sperm AR but not on capacitation	Not assessed	Thérien [70]
Bovine	Frozen	P4 ↟ AR	Intracellular pathways involving protein kinase C and the voltage-dependent calcium channel.	Córdoba [71]
Porcine	Fresh	P4 ⇈ the capacitation process but does not affect AR directly	Local counter-current systems	Barboni [72]

**Key: **↟** **induce;** ⇞⇟ **no influence;** **⇈** **increase;** **⊗** **Inhibit;** **⇊** **decreases****

### 4.1 *In vitro* animal models using oviductal epithelial cells to study sperm functionality

Oviductal epithelial cells were obtained from porcine, bovine, and ovine oviducts, and E2 and P4 were added to sperm in the OECs (Table 4). In the studies involving porcine sperm and OECs samples [48,49,52], it was reported that the binding of fresh boar sperm to the OECs may be high when steroid levels in the oviduct are at levels similar to the estrus phase (i.e., high E2 phase). This implies that high E2 is essential for the binding ability of sperm to the OECs. Furthermore, [21,22,50,52] demonstrated that P4 treatment promotes fresh boar and frozen bull sperm release from the OECs, while P4 at 100ng/ml increased sperm capacitation and fertilizing ability [37]. Besides, [23] found that 10, 100, and 1000ng/ml P4 induced the release of 32–47% bound frozen bull sperm from the OECs. Therefore, a desired P4 concentration is required to induce sperm capacitation important for the release of sperm from the OECs and subsequent sperm fertilizing ability.

**Table 4:** Description of the *in vitro* studies using oviductal epithelial cells to assess sperm functionality following steroid hormone supplementation.

Species	Semen type	Sample size for oviducts and semen collection	Dose of steroid hormone	First Author
Bovine	Frozen	Not Reported for Oviducts, 1 bull	1µg/ml E2	Boquest [53]
Porcine	Fresh	Not Reported	70pg/ml E2	Suarez [48]
Porcine	Fresh	15-20 Oviducts, 5 boars	80, 800 (nM) P4	Sharif [21]
Porcine	Fresh	15-20 Oviducts, 5 boars	80, 800 (nM) P4	Machado [22]
Bovine	Frozen	Not Reported for Oviducts, 3 bulls	5, 8, 10, 50, 500 (nM) P4 1, 10, 50, 80 (µM)	Romero-Aguirregomezcorta [50]
Porcine	Not Reported	Not Reported	4ng/ml E2 + 100ng/ml P4	Cimini [37]
Bovine, Ovine	Cooled	20 bovine Oviducts, 3 Rams	290pg/ml E2 + 6, 85ng/ml P4	Alfradique [51]
Bovine	Frozen	2 Oviducts, 3 bulls	1, 100(pg/ml), 100ng/ml E2 + 10, 100, 1000 (ng/ml) P4	Lamy [23]
Porcine	Fresh	4 Oviducts, Not Reported for boars	100ng/ml E2 + 100ng/ml P4	Bureau [52]
Bovine	Frozen	Not Reported for Oviducts, 5 bulls	1µg/ml E2 + 1 µg/ml P4	Lapointe [54]
Porcine	Fresh	Not Reported for Oviducts and boars	2,70(pg/ml) E2 + 0.5ng/ml P4	Raychoudhury [49]

In the study by [23], E2 at concentrations higher than 100 pg/ml inhibited the P4-induced release of bound sperm in a dose-dependent manner. Indeed, high E2 concentrations are required for facilitating sperm binding to the oviduct and to enhance sperm viability. Strikingly, pretreatment of E2 at 1 µg/ml increased the ability of OECs to prolong frozen bull sperm motility after 18 hr, and 36 hr in isthmic and ampullary cultures without affecting fertilization rates [53]. Additionally, E2 and P4 enhanced the ability of isthmic and ampullary cultures to prolong the motility and survival of both fresh and frozen bull sperm [53,54] in a hormone-dependent manner.

### 4.2 Mechanisms of action of estrogen and progesterone on sperm function in the sperm-oviductal epithelium co-cultures in the in vitro studies

The sperm-specific mechanisms of E2 on sperm physiology in sperm-OECs co-cultures are not yet fully understood, as evidenced by our review (Tables 2 and 3). Studies that assessed the E2 supplementation impact on sperm function in the sperm-OECs co-cultures did not investigate the specific mechanisms of action of E2. Therefore, further research is needed to elucidate the pathways and interactions through which E2 influences sperm behavior in the sperm-OECs co-cultures, as well as its role in regulating molecule secretion by the OECs. Moreover, different doses of E2 were used in the studies, and these variations of E2 may alter protein and amino acid secretion, gene expression, and ultimately affect sperm behavior differently [73,74]. In addition, E2 was found to inhibit the P4-induced sperm release from the OECs, in these studies, the specific mechanisms of action of E2 were not assessed [23,52].

Progesterone on the other hand, has been shown to facilitate the release of sperm from the OECs through active cation channels of sperm (CatSper) and proteasomal breakdown [21,22,50]. Besides, sperm hyperactivation was shown to be induced following P4 treatment by non-genomic pathways by stimulating CatSper [50].

### 4.3 Sperm functionality in the *in vitro* animal models devoid of oviductal epithelial cells

The species used in these studies included mice, rats, ovine, bovine, porcine, monkeys, and hamsters. One study used sperm samples from multiple species namely; mice, bovine, and porcine, while three studies used mouse sperm exclusively. Bovine sperm was used in six studies, golden hamster and boar sperm were each employed in four studies. Additionally, single studies incorporated sperm from rats, monkeys, and ovine species in their culture experiments (Table 5).

**Table 5:** Description of the *in vitro* studies without oviductal epithelial cells to assess sperm functionality after steroid hormone supplementation.

Species	Semen type	Sample size for semen collection	Dose of steroid hormone	First Author
ICR Mice	Fresh	Not Reported	10, 20, 40 (ng/ml) P4	Suzuki [27]
Rats	Fresh	130 Rats	10, 20, 40 (ng/ml) P4	Miyazawa [56]
Bovine	Fresh	60 Bulls	1mg/ml P4	Denisenko [57]
Rhesus Monkey	Fresh	5 Monkeys	1, 5, 10 (µM) P4	Sumigama [44]
Golden Hamsters	Fresh	Not Reported	20ng/ml P4	Fujinoki [62]
Golden Hamsters	Fresh	Not Reported	10, 20, 40 (ng/ml) P4	Noguchi [67]
Bovine	Frozen	4 Bulls	0, 0.1, 1.0, 10 (µg/ml) P4	Lukoseviciute [69]
Bovine	Fresh	4 Bulls	0.01-10 µg/ml P4	Thérien [70]
Bovine	Frozen	Not Reported	1µM P4	Córdoba [71]
Porcine	Fresh	7 Boars	1, 10,100, 1000 (nM) P4	Barboni [72]
ICR Mice	Fresh	Not Reported	2, 20 (ng/ml) and 2, 20, 200 (pg/ml) E2 + 20ng/ml P4	Fujikura [55]
Ovine	Fresh	8 Ram	1µM E2 + 1µM P4	Gimeno-Martos [28]
Porcine	Fresh	4 Boars	0.003,0.033,0.333 (µg/ml) E2 + 0.033, 0.333, 3.333(µg/ml) P4	Ayala [58]
Golden Hamster	Fresh	4 Hamsters	1,2,10,100, 200,400 (pg/ml) 1,10,20 (ng/ml) E2 + 10,20,40(ng/ml) P4	Fujinoki [61]
Bovine, ICR Mice, Porcine	Frozen, Fresh	3 mice, Not Reported for Bovine and Porcine	0.001-100µM E2 + 0.001–100µM P4	Ryu [60]
Golden Hamster	Fresh	Not Reported	2, 20ng/ml E2, 2, 20 200pg/ml E2, 2, 20, 200fg/ml E2 + 20ng/ml P4	Fujinoki [64]
Bovine	Frozen	4 Bulls	1µg/ml E2 + 1 µg/ml P4	Lukoseviciute [68]
Porcine	Fresh	6 Boars	10µg/ml E2	Gray [59]
BALB/c Mice	Fresh	Not Reported	0.2, 2, 20, 200 (ng/ml) E2	Sebkova [63]
Porcine	Not Reported	Not Reported	1nM-100µM E2	Ded [66]
Bovine	Fresh	3 bulls	2,4,8 (µg/ml) E2	Ciftci [65]

### 4.4 Use of progesterone alone in culture medium without oviductal epithelial cells to assess sperm functionality

Ten studies used P4 alone to assess sperm functionality in a culture medium (Table 5). P4 was shown to affect sperm hyperactivation in the studies using golden hamster, rat, and macaque sperm in which sperm hyperactivation at 20ng/ml and 10µM was observed [27,44,56,62,67] and increased IVF success rate after incubation [27,72] but 20ng/ml [56], 10 and 40ng/ml P4 did not affect IVF success rates [27]. This suggests that, for hyperactivation to be achieved in various animal species, a desired concentration of P4 is required before fertilization occurs.

On the other hand, P4 in a dose-dependent way facilitated capacitation and improved the fertilizing ability of boar sperm following co-incubation with oocytes [72] and increased bull sperm capacitation dependent on dose, in which a maximum effect was observed at 10 µg/ml but displayed no effect on the AR and plasma membrane stability following co-incubation with frozen bovine sperm [69]. Additionally, [44] demonstrated that P4 accelerates the completion of the capacitation of monkey sperm. Furthermore, P4 affected AR in a concentration-dependent manner in the monkey and bovine sperm [44,57,70,71]. These findings show that sperm from various animal species undergo capacitation and AR following P4 supplementation. Nonetheless, the specific mechanisms of action in these species need to be clarified.

### 4.5 Use of both progesterone and estrogen and estrogen alone in culture medium in the absence of oviductal epithelial cells to assess sperm functionality

A combination of E2 and P4, and E2 alone (Table 5) effects on sperm functionality in culture media were assessed in a few studies. Seven studies used a combination of E2 and P4, while four studies used E2 alone. Our synthesis shows that P4 promoted mouse sperm hyperactivation at 20ng/ml [27], and hamster sperm hyperactivation, which was suppressed by E2 at 1pg/ml, 200pg/ml, and 2–20ng/ml [27,61,64], similar to the findings observed in sperm-OECs co-cultures, even though different doses were used in these studies. Notably, P4 at 20ng/ml promoted the IVF success rate of mouse oocytes after co-incubation [27,55]. However, this effect was inhibited when 20ng/ml of E2 was introduced [55].

Likewise, [68] reported that P4 causes capacitation and AR in bovine and mouse sperm that can be alleviated by E2. Interestingly, E2 stimulated capacitation of boar and mouse sperm at different doses [60,66]. Conversely, [63] found that increasing E2 can significantly lower mouse sperm capacitating ability. These species differences in the effects of E2 and P4 warrant further clarification to deduce mechanisms. Strikingly, [59] found that E2 had no significant effect on the overall motility and progressive motility of freshly harvested porcine spermatozoa. Nonetheless, [65] found that at a dose of 2 µg/ml, E2 enhanced total bovine sperm motility as well as forward progressive motility. Surprisingly, sperm viability was unaffected by lower doses of 2–4 µg/ml, but sperm viability was decreased by 8 µg/ml of E2 [65]. Additionally, activation of AR via membrane receptors was reported after exposing ram sperm to P4 and E2 [28]. In yet another study, steroid hormone supplementation subtly affected the motility parameters of boar sperm [58].

### 4.6 Mechanisms of action of estrogen and progesterone on sperm function in the culture media without oviductal epithelial cells in the *in vitro* studies

This review indicates that many studies have been conducted *in vitro* without sperm-OEC co-cultures. In these studies, E2 inhibited P4-induced sperm hyperactivation via E2 receptors [55]. Indeed, P4 and E2 induced AR, capacitation via P4 receptor and E2 (ERα and ERβ) receptors [27,49,55] and intracellular pathways involving protein kinase C and voltage-dependent calcium channels [59,69,72]. Moreover, P4 increased hyperactivation through inositol 1,4,5-triphosphate, protein kinase C, and protein kinase A signals [62]. Furthermore, [56] found that P4 works via membrane receptors regulated by phospholipase C, transmembrane adenylate cyclase, and protein kinase A. Interestingly, P4 increased sperm hyperactivation via the non-genomic signal [67], and P4-enhanced hyperactivation was suppressed by E2 via inhibition of tyrosine phosphorylation through the non-genomic pathways [64]. In addition, E2 reduced sperm capacitation via membrane receptors and tyrosine phosphorylation [63]. Similar to the studies conducted with sperm-OECs cultures, the mechanisms underlying E2’s effect on sperm behavior *in vitro,* in the absence of OECs, have received limited attention. Strikingly, some studies showed that E2 stabilized the sperm plasma membrane [68], increased capacitation and AR in mouse sperm, and increased capacitation in porcine sperm at a low dose while increasing AR in porcine sperm at a high concentration, and [60] induced capacitation of boar sperm [66]. Besides, E2 affected motility measurements [58]. Regardless of these findings, the mechanisms of action of E2 remain unclear.

## 5. Discussion

This systematic review aimed to summarize the *in vitro* studies that have been carried out to study sperm function following supplementation with E2 and P4. Most of these studies demonstrated that dose-dependent P4 supplementation increases sperm release from the OECs and stimulates AR, capacitation, and hyperactivation. The differences in the effects of P4 and E2 in the various experiments could be due to heterogeneity in the methods used, such as obtaining sperm samples from different animal species with different genetic makeups, different evaluation times, and age differences that went unreported in many cases probably due to difficulties in tracing the animal ages at the slaughterhouses when collecting the oviducts. Interestingly, both the region of the oviduct and the stage of the estrous cycle from which the oviducts are collected for OECs processing affected bull sperm motility following steroid supplementation [54]. Consistently, luteal bovine OECs showed lower progressive and total motility than the negative controls [51]. However, the binding of ram sperm to the bovine OECs is not affected by the estrous cycle stage of the oviducts from which the OECs are extracted [51]. In agreement with [50,51] reported that the binding of bull sperm to the OECs is not affected by the stage of the estrous cycle. These findings highlight the need for further investigation into species-specific and cross-species mechanisms underlying these similarities and differences.

In studies using culture media without OECs, it was demonstrated that P4 supplementation of sperm increases the sperm AR, and capacitation. Strikingly, E2 exhibited dose-dependent effects on bull sperm function in culture media lacking OECs. At higher concentrations, E2 reduced bull sperm survival, likely due to increased oxidative stress and mitochondrial dysfunction [75]. In contrast, lower doses increased sperm motility and acrosome integrity [65], potentially by modulating calcium signaling pathways and stabilizing membrane fluidity [76]. Furthermore, [53] reported that bovine oviducts provide an optimal environment for the survival of sperm when the levels of E2 are high. Additionally, the decreased sperm survival in culture media without OECs may be due to a lack of beneficial molecules compared to the situation in the sperm-OECs co-cultures, in which OECs secrete useful molecules in the presence of E2, thereby increasing sperm binding to the sperm reservoir and survival [23]. Notably, it was reported in several studies that P4 affects sperm functionality through intracellular pathways involving voltage-gated calcium channels [21,22,50,70]. However, there is a need for further research to investigate the species-specific mechanisms of action of E2 and P4 supplementation on sperm physiology in the OECs and their effects on the success of ARTs.

This review demonstrated that the effects of E2 and P4 on sperm functionality varied with dose in studies using sperm-OECs co-cultures and studies not using OECs. Thus, both *in vitro* and *in vivo,* E2 and P4 need to reach a certain concentration to facilitate sperm binding or release from the sperm reservoir, induce an AR, and promote sperm capacitation in animals. In post-pubertal Holstein bulls, a similar positive correlation between E2 and sperm motility was found *in vivo* [65], agreeing with some of our findings in this review. Besides, high rates of ovum fertilization require efficient transportation of sperm from the cervix to the oviducts; thus, increased motility is a key factor influencing fertilization [65].

Our synthesis shows that E2 enhanced the binding ability of frozen bull and fresh boar sperm to the OECs. This suggests that E2’s role in sperm reservoir formation may be conserved across species and could potentially be leveraged in IVF programs in several animal species to regulate sperm competition, optimize fertilization efficiency, and improve reproductive outcomes. Moreover, this binding to the OECs and subsequent release by P4 has been shown to have a beneficial effect on IVF outcomes [23,77]. Interestingly, the cleavage and blastocyst rates obtained following fertilization of oocytes with sperm released from the bovine OECs following P4 supplementation were higher [23]. Likewise, the best IVF outcomes were obtained when porcine OECs were pre-treated with P4 [37]. Besides, it has been reported that only spermatozoa with intact membranes, high-quality chromatin, and superior morphology can bind to the OECs [77], thus binding to the OECs can be enhanced by E2, and a subpopulation of this superior bound sperm [13] can be released from the OECs by P4 supplementation and used subsequently in IVF programs to improve outcomes. Notably, in culture media alone without OECs, P4 has been studied for its impact on embryo quality in mice and porcine, yet key parameters require further clarification. In a concentration-dependent manner, P4-induced mouse and boar sperm hyperactivation, leading to an increase in the percentage of two-cell embryos and an increase in the number of penetrated sperm per oocyte than controls, thereby affecting the IVF success rates [27,55,72]. Conversely, when rat sperm and cumulus-oocyte complexes were co-incubated with P4, the number of two-cell embryos showed an upward trend, but the effect was not statistically significant [56]. Strikingly, E2 in mice suppressed the P4-induced increase in IVF success rate in a dose-dependent manner. This implies that steroid hormones competitively regulate hyperactivation and IVF [55]. Future studies should focus on explicitly assessing the division and blastocyst rates, percentage and type of cell fragmentation, visualization of nuclei and multinucleation, zona pellucida morphology, and compaction degree across species to clarify the effects of steroid hormone supplementation on embryo development and IVF outcomes if our comprehension is to be improved.

It has been previously reported that extracellular Ca2 + enters cells through several channels, including voltage-activated channels and store-operated channels, while intracellular Ca2 + is released by receptor-operated channels [78]. Indeed, E2 and P4 interact with sperm and OECs via their receptors [28,55,59]. This initiates genomic signaling, in which the receptor-hormone complex functions as a transcription factor, controlling gene expression and regulating the oviductal environment [27,56,79,80]. Non-genomic signaling involves the activation of the CatSper channel on sperm, which causes calcium influx. As a result, this improves sperm hyperactivation and motility, which are essential for fertilization [21,22,50]. E2 and P4 signaling pathways play key roles in preparing the endometrium for embryo implantation *in vivo* [81,82]*.* Besides, fertility treatments such as IVF, frequently include hormone therapy to optimize these pathways and boost success rates. These insights not only advance current fertility treatment options but also pave the way for novel approaches to complex reproductive challenges.

## 6. Limitations

A key limitation identified in our review is the considerable heterogeneity in methodologies among the included studies. The absence of standardized protocols for steroid hormone supplementation restricts cross-study comparisons and undermines the generalizability of the findings. Furthermore, several experiments lacked clear rationale or justification for the dosages used, making it difficult to align these *in vitro* conditions with physiologically relevant *in vivo* conditions. Our synthesis also reveals a notable gap in the studies assessing steroid hormone supplementation in the context of sex-sorted sperm. These studies were excluded due to the absence of eligible articles meeting our predefined inclusion criteria, which focused specifically on steroid hormone-supplemented *in vitro* experiments. This absence may reflect limitations in database coverage, a paucity of studies within this niche domain, or the stringency of our selection parameters. Additionally, our review underscores a noticeable species bias. For instance, equine studies were excluded from this review, primarily due to the restricted applicability of this species within conventional IVF protocols. Although equines are not commonly utilized in standard IVF procedures, their unique reproductive physiology presents unique opportunities for advancing ARTs. Consequently, there remains a need for species-specific *in vitro* investigations to better understand reproductive mechanisms and improve ART outcomes in equines.

## 7. Implications for future research

Several studies reported the influence of steroid hormones on sperm functionality without any scientific justification for the selected doses. Therefore, future research should prioritize methodological standardization. Additionally, future studies should compare sperm function following supplementation with physiological and oviductal fluid concentrations of the steroid hormones to ascertain the differences across animal species. Sperm-oviduct interaction investigations after steroid hormone supplementation to compare effects across different animal species, including equines and wildlife species on IVF rates may uncover conserved mechanisms that may aid in improving outcomes of ARTs in these animal species. Similarly, elucidation of species-specific mechanisms of action of E2 supplementation on sperm behavior in culture media with or without OECs, and to clarify the underlying pathways responsible for its suppressive effects on sperm hyperactivation, is needed. To better understand the sperm fertilizing ability in mammalian species *in vivo* events through *in vitro* techniques, more research is needed to understand how steroid hormone supplementation modulates the secretion of molecules from the OECs and their effect on IVF rates. Most previous *in vitro* studies assessing the impact of E2 and P4 supplementation used conventional semen to assess sperm oviductal interactions and sperm function in culture media. With the current increase in the use of sorted- sperm, we suggest more use of sex-sorted semen in the *in vitro* experiments following E2 and P4 supplementation to enhance our understanding of the differences in the X and Y-bearing sperm interaction with the oviductal epithelium to deduce molecular, hormonal, and biochemical factors that may aid in improving the fertilizing ability of sex-sorted semen in ARTs.

## 8. Conclusion

This systematic review highlights the dose-dependent, species-specific effects of E2 and P4 on sperm functionality in *in vitro* animal models. P4 promotes key fertilization-related processes such as sperm hyperactivation, capacitation, and AR, primarily through CatSper channel activation, while E2 modulates these responses in a more complex manner, often inhibiting P4-induced sperm hyperactivation and release from OECs across species. These findings underscore the dynamic hormonal interplay influencing sperm physiology and point to significant variation based on species and hormone concentrations.

The practical relevance to ARTs lies in the opportunity to refine sperm handling protocols by leveraging hormone-driven enhancements in motility and fertilizing capacity. Developing *in vitro* culture systems with precise hormonal supplementation may offer a more biologically attuned approach to improving ART outcomes. To advance clinical translation, future research should focus on clarifying the molecular mechanisms underpinning E2’s modulatory effects, standardizing hormone dosing across species, and incorporating physiologically relevant models that reflect the complexity of sperm-oviduct interactions. These directions will help bridge *in vitro* findings with *in vivo* applicability and optimise ART methodologies accordingly.

## Supporting information

S1 Data Figure2_raw data used to generate graph in Figure 2. (XLSX)

S2 Data Figure3_raw data used to generate figure 3. (XLSX)

S3 Data Figure4 raw_data used to generate figure 4. (XLSX)

S4 Data Figure5_raw data used to generate figure 5. (XLSX)

S1 File PRISMA Checklist. (DOCX)
